# Hallmarks of progeroid syndromes: lessons from mice and reprogrammed cells

**DOI:** 10.1242/dmm.024711

**Published:** 2016-07-01

**Authors:** Dido Carrero, Clara Soria-Valles, Carlos López-Otín

**Affiliations:** Departamento de Bioquímica y Biología Molecular, Facultad de Medicina, Instituto Universitario de Oncología (IUOPA), Universidad de Oviedo, Oviedo 33006, Spain

**Keywords:** Ageing, Progeria, Rejuvenation, iPSCs

## Abstract

Ageing is a process that inevitably affects most living organisms and involves the accumulation of macromolecular damage, genomic instability and loss of heterochromatin. Together, these alterations lead to a decline in stem cell function and to a reduced capability to regenerate tissue. In recent years, several genetic pathways and biochemical mechanisms that contribute to physiological ageing have been described, but further research is needed to better characterize this complex biological process. Because premature ageing (progeroid) syndromes, including progeria, mimic many of the characteristics of human ageing, research into these conditions has proven to be very useful not only to identify the underlying causal mechanisms and identify treatments for these pathologies, but also for the study of physiological ageing. In this Review, we summarize the main cellular and animal models used in progeria research, with an emphasis on patient-derived induced pluripotent stem cell models, and define a series of molecular and cellular hallmarks that characterize progeroid syndromes and parallel physiological ageing. Finally, we describe the therapeutic strategies being investigated for the treatment of progeroid syndromes, and their main limitations.

## Abstract (precis)

**Summary:** This Review defines the molecular and cellular hallmarks of progeroid syndromes according to the main cellular and animal models, and discusses the therapeutic strategies developed to date.

## Introduction

The physiological deterioration that accompanies ageing constitutes a major risk factor for the development of human pathologies, such as cancer, cardiovascular disorders and neurodegenerative diseases (Kennedy et al., 2014). Key molecular hallmarks of the ageing phenotype include telomere attrition, genomic instability, loss of proteostasis, epigenetic alterations, mitochondrial dysfunction, deregulated nutrient sensing, stem cell exhaustion, cellular senescence and altered intercellular communication (Lopez-Otin et al., 2013). At the macromolecular level, ageing is characterized by the development of wrinkles, greying and loss of hair, presbyopia, osteoarthritis and osteoporosis, progressive loss of fertility, loss of muscle mass and mobility, decreased cognitive ability, hearing loss, and a higher risk for the development of cancer and heart diseases, among other features (López-Otín et al., 2013).

Progeroid syndromes are a group of very rare genetic disorders that are characterized by clinical features that mimic physiological ageing, such as hair loss, short stature, skin tightness, cardiovascular diseases and osteoporosis. Consequently, they constitute a relevant source of information to understand the molecular mechanisms involved in normal ageing. Progeroid disorders do not show differences in prevalence depending on sex or ethnic origin, and appear at an early age, mainly due to defects in the nuclear envelope and DNA repair mechanisms (Gordon et al., 2014). Affected individuals die at a young age, usually as a consequence of cardiovascular problems and musculoskeletal degeneration.

In this Review, we classify human progeroid syndromes into two main groups according to the mechanisms that underlie the disease. Next, we discuss recent findings in the study of progeroid syndromes, achieved through the use of cellular and animal models. On the basis of these findings, we propose nine candidate hallmarks of premature ageing, and highlight their similarities with those described for physiological ageing. These proposed hallmarks recapitulate the most remarkable characteristics of progeroid syndromes and define the mechanisms underlying their pathogenesis, which could provide ideas for future studies on both physiological and pathological ageing. Finally, we review different therapeutic strategies developed for the treatment of these rare but devastating diseases.

## A classification system for human progeroid syndromes

All progeroid syndromes are characterized by similar clinical features (Table 1), but their underlying mechanisms can vary depending on the mutated gene and the pathway that is consequently altered. Below, we have classified progeroid syndromes into two general categories based on the molecular pathway involved. The first group includes those syndromes caused by alterations in components of the nuclear envelope, such as Hutchinson-Gilford progeria syndrome (HGPS), Néstor-Guillermo progeria syndrome (NGPS), atypical progeria syndromes (APSs), restrictive dermopathy (RD) and mandibuloacral dysplasia (MAD). The second group consists of progeroid syndromes induced by mutations in genes involved in DNA-repair pathways, such as Werner syndrome (WS), Bloom syndrome (BS), Rothmund-Thomson syndrome (RTS), Cockayne syndrome (CS), xeroderma pigmentosum (XP), trichothiodystrophy (TTD), Fanconi anaemia (FA), Seckel syndrome (SS), ataxia telangiectasia (AT), ataxia telangiectasia-like disorder (ATLD), cerebroretinal microangiopathy with calcifications and cysts (CRMCC), and Nijmegen breakage syndrome (NBN). A subcategory of this group comprises dyskeratosis congenita (DC) and Hoyeraal-Hreidarsson syndrome (HHS), linked to mutations in components of the telomerase complex (see Box 1 for a glossary of terms) that cause telomere attrition.

**Table 1.**
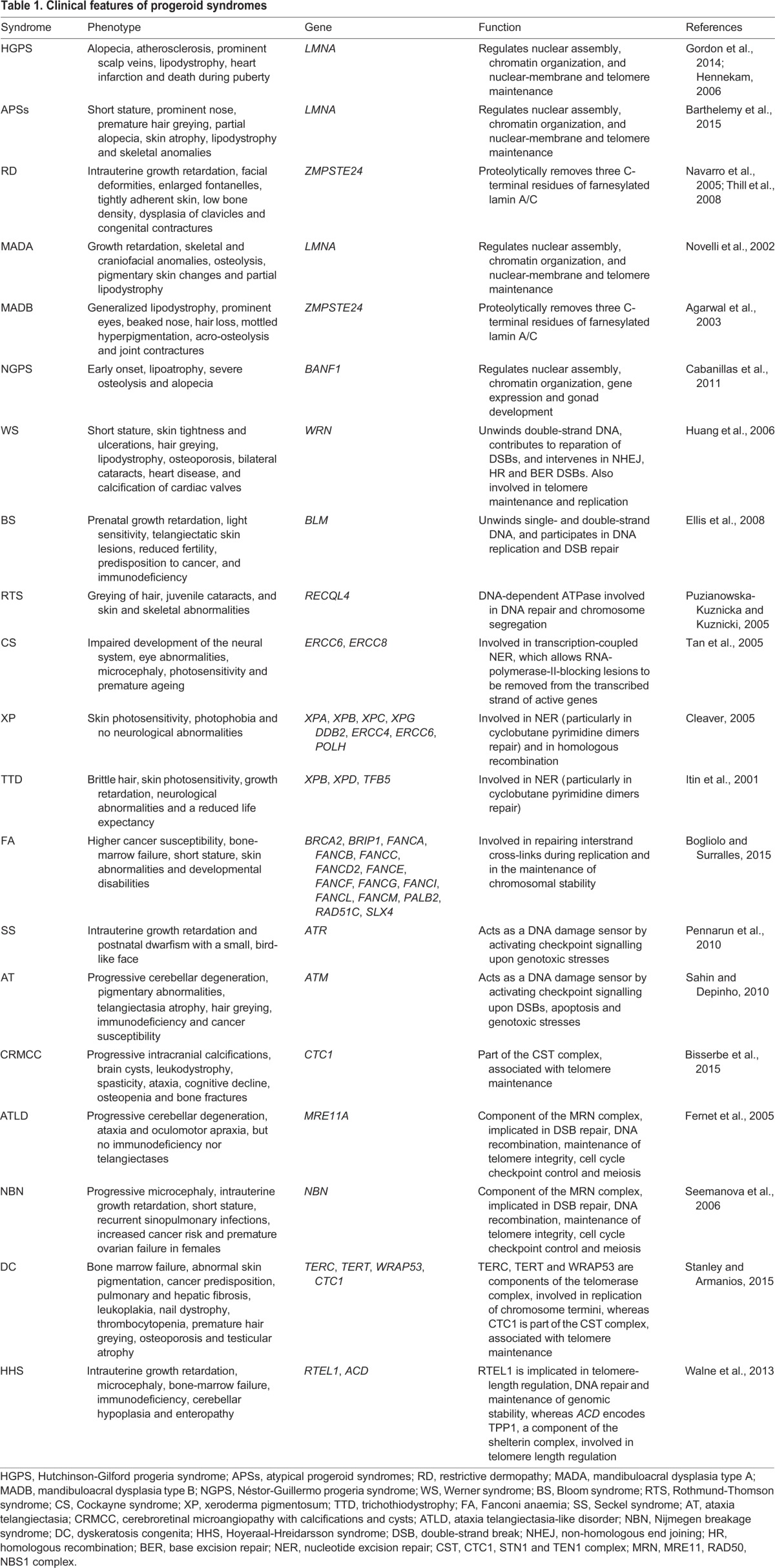
**Clinical features of progeroid syndromes**

**Box 1. Glossary**

**Emerin:** protein encoded by the *EMD* gene. Present in the inner nuclear membrane in vertebrates, and highly expressed in cardiac and skeletal muscle. Mutations in emerin cause X-linked recessive Emery-Dreifuss muscular dystrophy, cardiac conduction abnormalities and dilated cardiomyopathy.

**Cryptic splice site:** splice sites that are usually dormant and can be activated by nearby mutations, often causing genetic diseases.

**CST complex:** a complex involved in termination of telomere elongation, composed of CTC1, STN1 and TEN1.

**Double-strand break (DSB) repair:** double-strand breaks (DSBs) can lead to genome rearrangements. Three mechanisms exist to repair DSBs: non-homologous end joining (NHEJ), microhomology-mediated end joining (MMEJ) and homologous recombination (HR).

**Interstrand crosslinks (ICLs):** highly toxic DNA lesions that prevent transcription and replication by inhibiting DNA strand separation.

**Multipotent cell:** progenitor cell that has the gene activation potential to differentiate into multiple, but limited, cell types.

**Nucleotide excision repair (NER):** DNA-repair mechanism that removes DNA damage induced by ultraviolet (UV) light, mostly thymine dimers and 6,4-photoproducts.

**Pluripotent cell:** stem cell that has the potential to differentiate into any of the three germ layers: endoderm, mesoderm and ectoderm.

**RecQ helicase family:** helicases are enzymes important in replication and genome maintenance that unwind paired DNA and translocate in the 3′-to-5′ direction.

**Shelterin complex:** protein complex that protects mammalian telomeres from DNA-repair mechanisms, in addition to regulating telomerase activity. Subunits of shelterin bind to telomeres and induce the formation of a t-loop, a cap structure that prevents DNA-damage-sensing machinery from mistakenly repairing telomeres. The absence of shelterin causes telomere uncapping and thereby activates damage-signalling pathways that can lead to NHEJ, HR, senescence or apoptosis. Shelterin has six subunits: TRF1, TRF2, POT1, RAP1, TIN2 and TPP1.

**Somatotropic axis:** consists of growth hormone (GH) and insulin-like growth factors (IGF-I and -II) together with their associated carrier proteins and receptors. Regulates metabolism and other physiological processes. Other hormones, such as insulin, leptin, glucocorticoids and thyroid hormones, modulate GH and IGF synthesis and availability.

**Telomerase complex:** ribonucleoprotein complex encoded by the *TERT*, *TERC*, *DKC1* and *TEP1* genes that enlarges telomeres by adding the polynucleotide ‘TTAGGG’ to their 3′ end thanks to its reverse transcriptase activity, so that they can protect the ends of the chromosomes from deterioration or from fusion with other chromosomes.

### Nuclear architecture instability and premature ageing

**Fig. 1.**
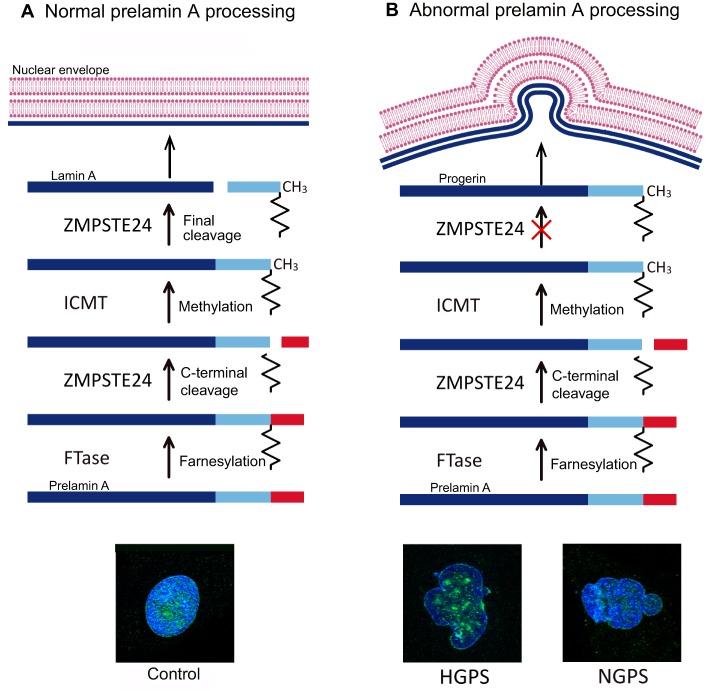
**Prelamin A physiological and pathological processing and maturation.** Processing of prelamin A in (A) normal cells, leading to the generation of mature lamin A and assembly of the normal nuclear envelope, and (B) HGPS cells, where the HGPS-associated mutation (G608G) in the gene encoding prelamin A, *LMNA*, activates a cryptic splicing site that results in the deletion of 50 amino acids slightly upstream of the C-terminus of prelamin A, encompassing the final cleavage site for ZMPSTE24 and leading to the accumulation of a toxic form of lamin A named progerin. This leads to disruption of the nuclear envelope, detectable as bulging or ‘nuclear blebbing’, which is shown in the representative images of HGPS and NGPS human fibroblasts illustrated below, in comparison to control cells. Lamin A/C (green) and DAPI (blue) staining is shown. Note that nuclear blebbing in NGPS cells is not due to the accumulation of progerin (see main text), and this image has been included purely to demonstrate the phenotype. FTase, farnesyltransferase; ICMT, isoprenylcysteine carboxyl methyltransferase; HGPS, Hutchinson-Gilford progeria syndrome; NGPS, Nestor-Guillermo progeria syndrome; ZMPSTE24, zinc metalloproteinase STE24.

The nuclear lamina is a highly regulated membrane barrier that separates the nucleus from the cytoplasm in eukaryotic cells, and contains lamins and other proteins involved in chromatin organization and gene regulation (Burke and Stewart, 2013) (Fig. 1). There are two major types of lamin proteins, the A-type, encoded by the gene *LMNA*, which includes lamins A and C, and the B-type, encoded by the genes *LMNB1* and *LMNB2*, and includes lamins B1, B2 and B3. Lamin A undergoes complex post-translational processing steps, such as farnesylation, cleavage by the zinc metallopeptidase STE24 (ZMPSTE24), carboxyl methylation by the isoprenylcysteine carboxylmethyltransferase (ICMT), and excision of the farnesylated residue (Fig. 1). Lamin A also interacts with many different proteins, such as the barrier to autointegration factor (BAF), to achieve mitotic and post-mitotic nuclear assembly (Jamin and Wiebe, 2015). Mutations in genes that encode nuclear-lamina proteins cause progeroid syndromes, such as HGPS, NGPS, APSs, RD and MAD (Agarwal et al., 2003; Barthelemy et al., 2015; De Sandre-Giovannoli et al., 2003; Eriksson et al., 2003; Navarro et al., 2005; Puente et al., 2011). In this section, we describe the main clinical features of progeroid syndromes caused by mutations in key elements of the nuclear envelope (Table 1).

#### Hutchinson-Gilford progeria syndrome

HGPS is the most prevalent and widely studied accelerated-ageing syndrome. Most cases of HGPS originate from a *de novo* heterozygous silent mutation in the *LMNA* gene (G608G). This mutation activates a cryptic splicing site (Box 1) that results in the deletion of 50 amino acids near the C-terminus of prelamin A, which encompasses the final cleavage site for CAAX prenyl protease 1 homolog (ZMPSTE24) to produce lamin A. This leads to the accumulation of a toxic protein called progerin, which disrupts the integrity of the nuclear envelope (Fig. 1) (De Sandre-Giovannoli et al., 2003; Eriksson et al., 2003). Individuals with HGPS display alopecia (hair loss), atherosclerosis, lipodystrophy, heart infarction and death during puberty (Table 1) (Gordon et al., 2014). Cells derived from these individuals present nuclear shape abnormalities known as ‘blebs’ (Fig. 1B) and shortened telomeres, and undergo premature senescence as a consequence of genome instability (Gonzalo and Kreienkamp, 2015). Progerin also accumulates during physiological ageing, reinforcing the parallels between normal and pathological ageing (Scaffidi and Misteli, 2006). Similar to aged individuals, HGPS individuals demonstrate vascular stiffening, atherosclerotic plaques and calcium dysfunction (Gerhard-Herman et al., 2012; Olive et al., 2010). Nonetheless, some basic features of ageing, such as the deterioration of the nervous system, the immune system deficits and the increased susceptibility to cancer, are not recapitulated in HGPS (Gordon et al., 2014). This is due to the low levels of prelamin A expression in the brain (Jung et al., 2012), and to the presence of a tumour protection mechanism mediated by bromodomain containing protein 4 (BRD4) in cells from individuals with HGPS (Fernandez et al., 2014) (discussed in more detail later).

#### Atypical progeria syndromes

Several lamin A mutations, including A57P, R133L and L140R, which are predicted to alter key protein-protein interaction domains, are associated with APSs. Individuals with APS show many of the clinical features of HGPS (Table 1), but their cells do not accumulate prelamin A or progerin (Barthelemy et al., 2015).

#### Restrictive dermopathy and mandibuloacral dysplasia

RD is a rare recessive condition caused by *ZMPSTE24* mutations that lead to the accumulation of lamin A precursors (Navarro et al., 2005). MAD is characterized by lipodystrophy and skeletal and metabolic abnormalities (Table 1). MAD with type A lipodystrophy (MADA) is induced by the homozygous R527H *LMNA* mutation, which leads to accumulation of prelamin A and changes in nuclear architecture (Novelli et al., 2002), whereas MAD with type B lipodystrophy (MADB) is caused by compound heterozygous mutations in *ZMPSTE24* (Agarwal et al., 2003).

#### Néstor-Guillermo progeria syndrome

NGPS is caused by a homozygous mutation (c.34G<A; p.Ala12Thr) in *BANF1* (barrier to autointegration Factor 1), which encodes the protein BAF, involved in chromatin organization, nuclear assembly and gene-expression regulation. Fibroblasts from NGPS individuals demonstrate reduced levels of BAF protein, aberrant nuclear morphology, namely blebbing (Fig. 1), and altered subcellular distribution of emerin (see Box 1) (Cabanillas et al., 2011; Puente et al., 2011). The A12T mutation also weakens the binding of BAF to DNA, which is crucial for its many roles in the cell, suggesting that this deficiency contributes to the cellular phenotype observed in NGPS (Paquet et al., 2014). NGPS shares many clinical features with HGPS (Table 1), but, for unknown reasons, lacks cardiovascular defects, which likely contributes to the longer lifespan of NGPS patients (Cabanillas et al., 2011).

### Defects in DNA repair, and premature ageing

**Fig. 2.**
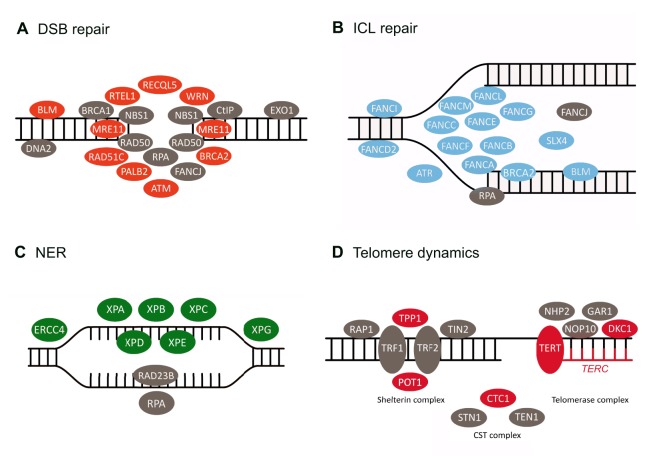
**Mutations in proteins involved in DNA repair lead to premature ageing syndromes.** Diagrams of the proteins involved in (A) double-strand break (DSB) repair, (B) interstrand cross-link (ISL) repair, (C) nucleotide excision repair (NER) and (D) telomere elongation and maintenance, including the shelterin complex, the telomerase complex and the CST (CTC1, STN1 and TEN1) complex (Box 1). Proteins encoded by genes mutated in progeroid syndromes are shown in orange, blue, green and red, whereas non-mutated proteins are shown in grey.

Damage to nuclear DNA is a direct cause of ageing and cancer, and a decline in DNA repair with age has been described (Vijg and Suh, 2013). Moreover, many progeroid syndromes are caused by defects in DNA repair (Childs et al., 2014; Vermeij et al., 2016). In humans, there are five RecQ helicases – proteins that repair double-strand breaks during DNA replication (Box 1), and maintain genome stability and telomere integrity (Bernstein et al., 2010). Mutations in three of them [*WRN* (Werner syndrome, RecQ helicase-like), *BLM* (Bloom syndrome, RecQ helicase-like) and *RECQ4* (RecQ helicase-like 4)] are associated with WS, BS and RTS premature-ageing syndromes, respectively. Other DNA-repair proteins altered in progeroid syndromes include the excision-repair cross-complementing (ERCC) family, the FA proteins and the XP proteins, which collaborate to enable DNA nucleotide excision repair (NER) and double-strand-break repair (Fig. 2; Box 1) (Vermeij et al., 2016). Mutations in genes encoding these and other proteins involved in DNA repair are responsible for different premature-ageing disorders, the clinical features of which are summarized below and in Table 1.

#### Werner syndrome

WS is caused by mutations in the *WRN* gene that result in the production of WRN truncated proteins (Yu et al., 1996). Individuals with this disorder display a range of premature-ageing symptoms (Table 1) and a high incidence of cancer that might be related to the chromosomal instability, telomere dysfunction and senescent phenotype exhibited by cells lacking WRN (Crabbe et al., 2007).

#### Bloom syndrome

BS is caused by mutations in the *BLM* gene, which encodes the RecQ helicase BLM. These mutations lead to the mislocalization of BLM protein or to the impairment of its helicase or ATPase domains (Machwe et al., 2005). This syndrome is characterized by immunodeficiency and predisposition to cancer, among other features (Table 1) (Ellis et al., 2008).

#### Rothmund-Thomson syndrome

RTS is caused by mutations in *RECQL4*, which encodes a helicase localized at telomeres and mitochondria (Croteau et al., 2012). This syndrome is mainly characterized by growth deficiency, skin abnormalities and increased susceptibility to cancer (Table 1) (Puzianowska-Kuznicka and Kuznicki, 2005). The loss of RECQL4 in fibroblasts of affected individuals leads to impaired DNA repair after oxidative stress and to growth arrest, which could explain the dwarfism associated with this disorder (Werner et al., 2006).

#### Cockayne syndrome, xeroderma pigmentosum and trichothiodystrophy

CS, XP and TTD are a group of related disorders associated with defects in NER (Box 1) (Marteijn et al., 2014). CS is a neurodegenerative disorder caused by mutations in the *ERCC6* and *ERCC8* genes, and is mainly characterized by neural abnormalities and growth failure (Table 1) (Tan et al., 2005). XP arises from mutation of any of many different genes, including *XPA*, *XPB*, *XPC*, *XPG*, *ERCC4*, *ERCC6*, *DDB2* and *POLH.* This syndrome is characterized by an impaired capacity to repair the damage caused by UV light, which leads to increased cancer susceptibility (Cleaver, 2005). TTD is another rare progeroid disorder generated by mutations in *XPB*, *XPD* or *TFB5*, and is characterized by skin photosensitivity, growth retardation and a reduced life expectancy (Itin et al., 2001).

#### Fanconi anaemia

Mutations in FA genes, which encode proteins that are involved in DNA repair, such as FANCA or BRCA2, can lead to FA disease. Individuals with FA exhibit a higher cancer susceptibility, bone marrow failure, short stature, skin abnormalities and developmental disabilities (Table 1) (Deakyne and Mazin, 2011). Cells from individuals with FA produce high levels of reactive oxygen species (ROS), which can damage telomeric regions (Uziel et al., 2008). This defect, together with an insufficient DNA-repair system, results in single-strand DNA breaks that induce accelerated telomere shortening. Mutant cells show shorter telomeres and an increase in chromosome-end fusions compared to normal controls, resulting in genomic instability and multinucleated cells (Bogliolo and Surralles, 2015).

#### Seckel syndrome

SS is caused by mutations in the *ATR* (ataxia telangiectasia and Rad3-related) gene, which codes for the serine/threonine kinase ATR. SS is characterized by intrauterine growth retardation and postnatal dwarfism (Table 1) (Shanske et al., 1997). ATR protein has a crucial role in preventing replicative stress, sensing DNA damage and consequently arresting the cell cycle, and in maintaining telomere integrity (Pennarun et al., 2010).

#### Ataxia telangiectasia

AT is caused by mutations in the *ATM* (ataxia telangiectasia mutated) gene, which encodes a serine/threonine kinase that is activated by DNA double-strand breaks. AT individuals show progressive cerebellar degeneration, pigmentary abnormalities, hair greying and increased cancer susceptibility (Table 1). ATM is involved in DNA-damage signalling, cell cycle arrest and telomere maintenance (Sahin and DePinho, 2010).

#### Dyskeratosis congenita and Hoyeraal-Hreidarsson syndrome

Telomere shortening is observed during normal ageing both in human and mouse cells, acting as a barrier to tumour growth and contributing to cell senescence (Armanios and Blackburn, 2012). Various premature ageing disorders, such as DC and HHS, are linked to mutations in components of the telomerase complex (Box 1). DC is a rare autosomal-dominant disorder caused by mutations in *TERC* (telomerase RNA component), *TERT* (telomerase reverse transcriptase), *WRAP53* (WD repeat component, antisense to TP53) or *CTC1* (CTS telomere maintenance complex component 1), among other genes (Stanley and Armanios, 2015). X-linked DC is caused by mutations in *DKC1* (dyskeratosis congenita 1) (Fig. 2) (Yoon et al., 2006). Individuals with DC present bone marrow failure, cancer predisposition, premature hair greying and osteoporosis (Table 1) (Stanley and Armanios, 2015). HHS is a very rare multisystem disorder that phenocopies DC but with an increased severity (Table 1) (Walne et al., 2013). Mutations responsible for HHS have been identified in *RTEL1* (regulator of telomere elongation helicase 1), which encodes a helicase that is essential for telomere maintenance, and *ACD* (adrenocortical dysplasia homolog), which encodes the shelterin TPP1 (Box 1) (Kocak et al., 2014; Walne et al., 2013).

The development of cellular and mouse models for the study of the progeroid syndromes described above has been extremely useful in order to elucidate the mechanisms underlying such diseases. The most relevant models are described in the following sections.

## Cellular models of progeroid syndromes

The generation of patient-derived induced pluripotent stem cells (iPSCs) in recent years has enabled researchers to study specific tissues affected by a disease and to discover new tissue-specific drugs (Studer et al., 2015; Tang et al., 2016). Recently, these approaches have also been used to study different progeroid syndromes and to identify barriers to reprogramming in normally aged and prematurely aged cells.

### iPSC generation from progeroid cells

iPSCs from several progeroid syndromes have been successfully generated and differentiated along multiple lineages (Table 2). However, progeria-derived iPSCs normally show reduced reprogramming efficiency in comparison to control cells despite being indistinguishable from normal iPSCs in other ways. In line with this, HGPS and NGPS iPSCs lack disease-specific features, such as nuclear blebs, epigenetic changes or, in the case of HPGS iPSCs, progerin expression, demonstrating the erasure of age-associated marks (Lo Cicero and Nissan, 2015; Soria-Valles et al., 2015a; Xiong et al., 2013). Once differentiated, however, the progeroid iPSC-derived cells show age-associated alterations, frequently mimicking the associated pathologies. These iPSC-derived models have been useful for studying the molecular mechanisms of HGPS and NGPS progerias and for screening drug candidates that could be used to treat these disorders (Pitrez et al., 2016; Soria-Valles et al., 2015b). Likewise, when WS fibroblasts are reprogrammed, their telomeres elongate, indicating that telomere function is restored (Shimamoto et al., 2014). The differentiation of WS iPSCs to mesenchymal stem cells (MSCs) results in a recurrence of premature senescence that is associated with telomere attrition. This phenotype can be rescued by the expression of hTERT or by knocking down p53 (Cheung et al., 2014). Another study, using iPSCs generated from CS fibroblasts, has demonstrated that lack of functional *ERCC6* increases cell death and ROS production, and upregulates *TP53* relative to normal cells (Andrade et al., 2012).

**Table 2.**
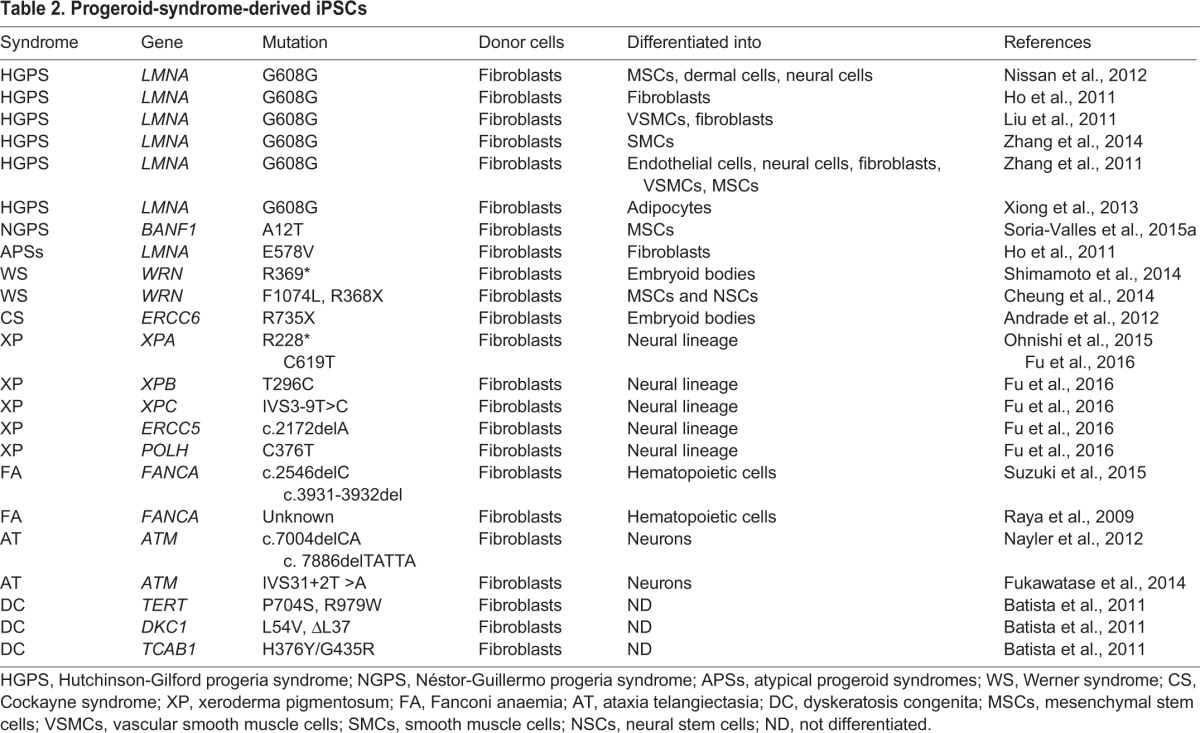
**Progeroid-syndrome-derived iPSCs**

XP iPSCs from patients with mutations in *XPA* have also been developed to produce *in vitro* models of neurological disorders (Fu et al., 2016). These iPSCs can be differentiated into neural cells, indicating that the expression of wild-type *XPA* alleles is not required for iPSC generation and differentiation (Ohnishi et al., 2015). Studies with DC-derived iPSCs have shown that, even in the undifferentiated state, these cells can exhibit substantially reduced telomerase levels or mislocalization of telomerase from Cajal bodies to nucleoli, which disables the telomere elongation that normally accompanies reprogramming (Batista et al., 2011). Notably, iPSCs from fibroblasts of individuals with *FANCA* mutations have an impaired ability to differentiate towards early hemoangiogenic progenitors, which indicates that the hematopoietic phenotype of individuals with FA originates from an early hematopoietic stage (Suzuki et al., 2015). Correction of the *FANCA* mutation increases reprogramming and differentiation capacities of the resulting cells (Raya et al., 2009).

AT-derived fibroblasts have also been reprogrammed, albeit at a reduced efficiency (Fukawatase et al., 2014). These iPSCs display hypersensitivity to ionizing radiation, alterations in DNA-damage signalling pathways, metabolic changes and cell cycle checkpoint defects. AT-derived iPSCs have been differentiated into functional neurons, providing a unique model for the study of AT-associated neurodegeneration (Nayler et al., 2012).

Surprisingly, iPSCs have not yet been derived from RD, MAD, RTS, TTD, BS or SS cells, nor from APSs. The reason for this might partly rest with the low reprogramming efficiency of progeroid cells owing to a series of reprogramming barriers that are starting to be elucidated, as discussed below.

### Barriers to reprogramming in ageing cells

Several features displayed by aged cells, such as genetic damage, telomere shortening and cell senescence, represent barriers that greatly reduce the efficiency of cell reprogramming. The reprogramming process is slow and inefficient in part because the forced expression of the Yamanaka factors is a stressful mechanism that activates apoptosis and cellular senescence via the upregulation of tumour suppressor proteins, including p53, p16 (INK4a) and p21 (CIP1) (Li et al., 2009). Accordingly, the inhibition of these factors improves reprogramming efficiency (Banito and Gil, 2010; Hong et al., 2009). Metabolic studies also indicate that inhibition of mammalian target of rapamycin (mTOR) notably improves the efficiency of iPSC generation (Chen et al., 2011), identifying mTOR as an important repressor of reprogramming.

Interestingly, NF-κB (nuclear factor kappa-light-chain-enhancer of activated B cells) activation constitutes another barrier to somatic cell reprogramming in normally and prematurely aged cells (Soria-Valles et al., 2015a). Accordingly, NF-κB inhibition significantly increased the reprogramming efficiency of NGPS and HGPS fibroblasts, and of fibroblasts from advanced-age donors. Progeroid fibroblasts also showed a noticeable overexpression of DOT1L (DOT1-like histone H3-K79 methyltransferase), induced by NF-κB. Consistent with this, DOT1L inhibition increased reprogramming efficiency of progeroid cells and physiologically aged fibroblasts. Remarkably, treatment of progeroid mice with DOT1L inhibitors extends their longevity, implicating DOT1L as a newly identified target for rejuvenation-based approaches (Soria-Valles et al., 2015b).

Cells from individuals with FA failed to be reprogrammed to iPSCs unless the defective FA gene was replaced (Muller et al., 2012; Raya et al., 2009). However, a recent study has revealed that E6 protein from human papillomavirus 16 (HPV16) rescues FA-derived iPSC colony formation via p53 inhibition (Chlon et al., 2014). Consequently, the FA pathway is required for reprogramming through p53-dependent mechanisms, emphasizing the importance of classical tumour suppressors as barriers for cell reprogramming in progeroid syndromes.

## Mouse models of progeroid syndromes

Mouse models have been widely used to explore the molecular mechanisms of ageing and progeroid syndromes. Thus, experiments using mice with an extended lifespan or with signs of premature ageing have helped to elucidate the basic processes that affect ageing (Table 3) (Lopez-Otin et al., 2013; Vanhooren and Libert, 2013).

**Table 3.**
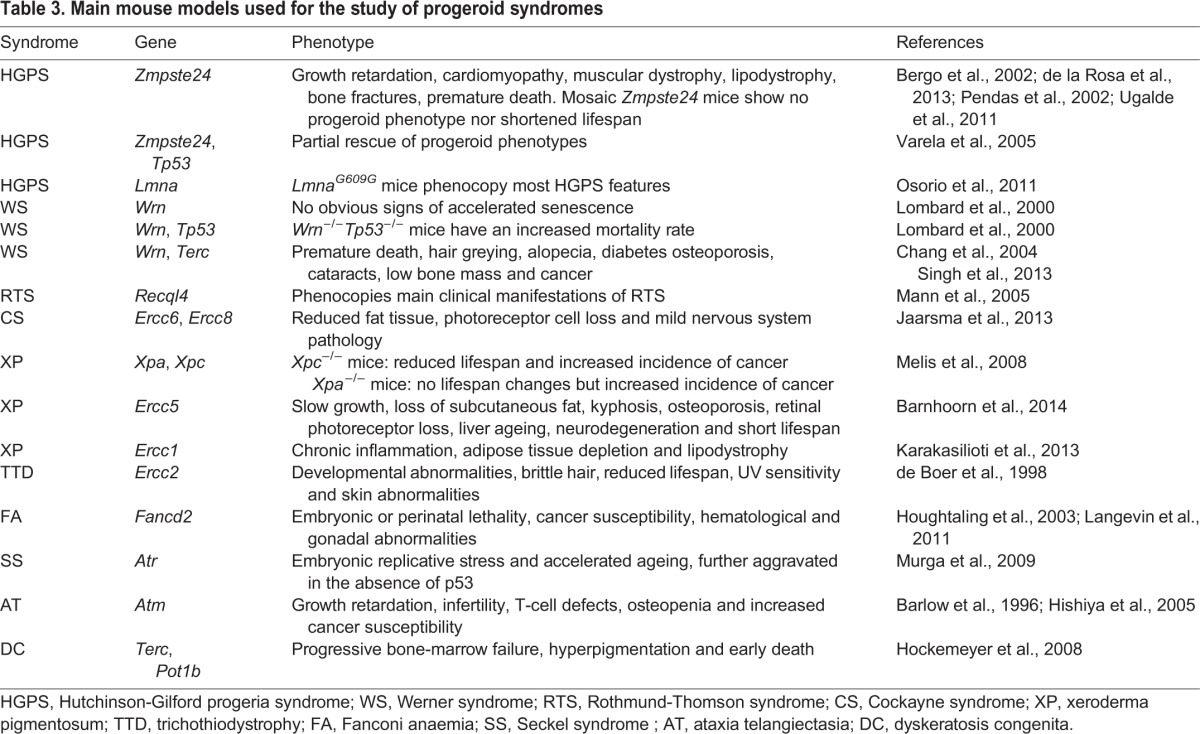
**Main mouse models used for the study of progeroid syndromes**

In 2002, Pendás et al. and Bergo et al., developed the first HGPS mouse model – *Zmpste24*-null (*Zmpste24^−/−^*) mice – which showed growth retardation, cardiomyopathy, muscular dystrophy, lipodystrophy and premature death (Bergo et al., 2002; Pendas et al., 2002). Studies of these mice demonstrated that the accumulation of farnesylated prelamin A damages the nuclear envelope, hyperactivates p53 signalling, and causes cellular senescence, stem cell dysfunction and a progeroid phenotype (Osorio et al., 2009; Varela et al., 2005). *Zmpste24^−/−^* mice are also defective in DNA repair (Liu et al., 2005), and in the p53-dependent upregulation of miR-29, which represses extracellular matrix genes (Ugalde et al., 2011). Extracellular matrix remodelling is a widely known mechanism that promotes renovation of adult tissues, and, consequently, repression of such genes impairs tissue restoration and promotes ageing (Gutierrez-Fernandez et al., 2015). However, this mouse model cannot be used to study the aberrant splicing of *LMNA* observed in individuals with HGPS, prompting the generation of a mouse strain that carries the HGPS mutation (*LMNA^G608G^*) (Osorio et al., 2011). These mice accumulate progerin and phenocopy the main clinical manifestations of human HGPS, such as shortened lifespan and bone and cardiovascular alterations. This model has been widely used to develop new therapeutic strategies for HGPS.

Other mouse models related to the lamin-A–Zmpste24 system have helped to elucidate why individuals with HGPS are not predisposed to cancer despite their elevated levels of DNA damage (Cau et al., 2014; Gordon et al., 2014). *Zmpste24* mosaic mice that contain both *Zmpste24*-proficient cells and *Zmpste24^−/−^* cells have revealed a lower incidence of invasive carcinomas compared to mice that are heterozygous for *Zmpste24* (de la Rosa et al., 2013). Parallel studies have described another cancer protective mechanism in HGPS cells involving suppression of cell proliferation and metastatic properties owing to an altered pattern of chromatin binding by the transcription regulator BRD4 (Fernandez et al., 2014). Other mouse models with *Lmna* mutations have been developed, although many of these do not fully recapitulate the progeroid phenotype observed in HGPS or APSs (Das et al., 2013; Poitelon et al., 2012; Varga et al., 2006). *Zmpste24^−/−^* mouse models have been used to study the molecular mechanisms of RD and MADB progeroid syndromes, although this mouse model does not fully recapitulate several features of these diseases (Osorio et al., 2009). In summary, many mouse models for laminopathies have been developed and studied in order to elucidate the mechanisms underlying the pathogenesis of these and related progeroid syndromes.

Several mouse models have also been created to mimic the clinical phenotypes associated with WS. *Wrn*-knockout mice have no obvious signs of accelerated senescence and fail to recapitulate clinical WS features (Lombard et al., 2000). However, *Wrn^−/−^Terc^−/−^* mice exhibit many of the key phenotypes, in support of a role for WRN in telomere maintenance (Chang et al., 2004). Mouse models of BS have also been developed, but their early lethality has hampered their utility for the *in vivo* study of this disease (Luo et al., 2000). Likewise, mice bearing the most common mutations found in RTS individuals have been generated. These mice show severe growth retardation, hair loss, dry skin and increased cancer susceptibility (Mann et al., 2005). Mutant mice have been developed for all CS-associated genes, most of which develop mild CS-like symptoms (Jaarsma et al., 2013). Mice mutant for *Xpa^−/−^* and *Xpc^−/−^* have also been reported. *Xpc^−/−^* mice demonstrate a reduced lifespan and an increased rate of lung cancer, whereas *Xpa^−/−^* mice show no lifespan alterations but exhibit a higher rate of liver carcinomas (Hosseini et al., 2015; Melis et al., 2008). *Xpg*-deficient mice also display many progeroid features, including loss of subcutaneous fat, osteoporosis, neurodegeneration and a short lifespan (Barnhoorn et al., 2014). Furthermore, DNA damage in mice carrying an Ercc1-Xpf DNA-repair defect triggers a chronic inflammatory response, leading to lipodystrophy (Karakasilioti et al., 2013). Finally, a mouse model of TTD recapitulates the human disorder, demonstrating developmental defects, brittle hair, UV sensitivity, skin abnormalities and reduced lifespan (de Boer et al., 1998).

There are several FA mouse models, all of which are characterized by embryonic or perinatal lethality, gonadal abnormality and associated infertility (Bakker et al., 2013). Nevertheless, most FA mouse models do not show any apparent hematological abnormalities, in contrast with humans with FA, who are affected by life-threatening anaemia. An exception to this is the *Fancp* mutant mouse, which has lowered numbers of white blood cells and platelets (Langevin et al., 2011). Hematopoietic stem cells (HSCs) from other FA mouse mutants (*Fancc*, *Fancd2* and *Fancg*) have a reduced repopulating ability and fail to maintain hematopoietic homeostasis under stress conditions (Bakker et al., 2013). *Fancd2*-, *Fancf-* or *Fancm-*deficient mice also exhibit an increased incidence of tumorigenesis (Bakker et al., 2013). The SS mouse model, characterized by ATR deficiency, shows high levels of replicative stress during embryogenesis and exhibits accelerated ageing, which is further aggravated in a *Tp53*-null background (Murga et al., 2009). AT mouse models recapitulate many of the characteristics observed in humans with AT, such as growth retardation, infertility, immune defects and increased susceptibility to lymphomas, although neurodegeneration is not observed (Barlow et al., 1996). Additionally, *Atm*-deficient mice show increased cancer susceptibility and a severe osteopenic phenotype, which is partly caused by a stem cell defect due to decreased expression of IGF, an important regulator of proliferation (Hishiya et al., 2005).

Mouse models of telomere dysfunction in progeroid syndromes have also been generated. The first mutant mouse with telomerase deficiency failed to recapitulate the clinical features of DC. However, when POT1b−a component of the shelterin complex that protects mammalian telomeres−is removed in mice (Box 1), these animals show clinical features of DC (Hockemeyer et al., 2008). Mouse models of APS, HHS, NBN, ATLD or CRMCC are yet to be developed.

## Hallmarks of progeroid syndromes

**Fig. 3.**
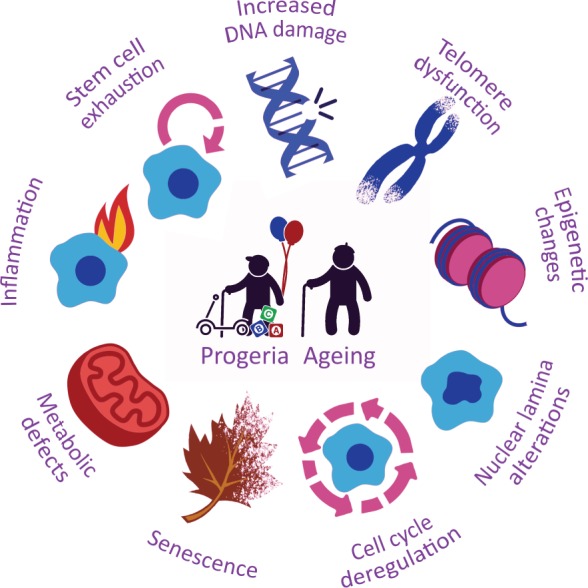
**The molecular and cellular hallmarks of progeroid syndromes.** These nine proposed hallmarks recapitulate the most remarkable features common to different progeroid syndromes and define the mechanisms underlying the pathogenesis of these diseases.

Based on the evidence obtained through experimentation with cellular and animal models, we propose a set of hallmarks for defining the main features of progeroid syndromes, and discuss their relatedness and differences with those of normal ageing (Fig. 3). Defining these molecular hallmarks could help in the design and interpretation of future studies of both physiological and premature ageing, and could also provide clinical benefit by guiding the diagnosis of or therapy for progeroid diseases.

### Increased DNA damage and defective DNA repair

Both physiologically aged and progeroid cells accumulate genomic damage throughout life (Vijg and Suh, 2013). Somatic mutations and other forms of DNA damage progressively compile within cells from both aged humans and model organisms, affecting essential genes and resulting in dysfunctional cells and impaired organismal homeostasis (Lopez-Otin et al., 2013). Many other age-related changes can also affect DNA repair mechanisms, leading to the accumulation of more genomic damage (Gorbunova et al., 2007). HGPS and RD progeroid cells are particularly susceptible to DNA damage induced by ROS or by ionizing radiation, and show a severely impaired capacity to repair DNA damage (Camozzi et al., 2014). Many progeroid syndromes also present repair defects owing to mutations in RecQ helicases (Box 1), or in proteins belonging to the ERCC and XP families (Bernstein et al., 2010; Marteijn et al., 2014).

### Telomere dysfunction

Telomere shortening is observed during normal ageing in both human and mouse cells, and has been linked to a decreased lifespan (Blasco et al., 1997). As discussed earlier, progeroid syndromes such as DC and HHS are caused by mutations in different components of the telomerase complex. Other ageing-related diseases, such as WS, FA, SS, AT, ATLD, NBN and CRMCC, are characterized by telomere dysfunction caused by mutations in genes involved in telomere maintenance and DNA repair (Armanios and Blackburn, 2012). HGPS cells also exhibit accelerated telomere shortening during proliferation in culture (Decker et al., 2009). Notably, the ectopic expression of telomerase in these cells increases their proliferation and lifespan by decreasing progerin-induced activation of the p53 and retinoblastoma (Rb) pathways (Kudlow et al., 2008). Additionally, the association of the shelterin TRF2 (telomere repeat-binding factor 2) with telomeric sequences is stabilized by its colocalization with A-type lamins. *LMNA* mutations lead to the impaired association of these lamins with TRF2, resulting in telomere loss (Wood et al., 2014). Furthermore, the ectopic expression of progerin in normal fibroblasts results in the accumulation of DNA damage at telomeres and in cell senescence (Cao et al., 2011). These studies suggest that telomere dysfunction and associated defective DNA repair contribute to genomic instability and premature senescence of cells in progeroid syndromes.

### Changes in epigenetics and chromatin structure

Chromatin remodelling plays a central role in the regulation of gene expression. As such, altered chromatin structure can lead to aberrant gene expression patterns, altering normal cellular functions and contributing to both normal and premature ageing (Chandra et al., 2015). Aged cells also accumulate epigenetic alterations that alter normal gene expression profiles (Beerman and Rossi, 2015). Accordingly, aged cells exhibit increased levels of histone modifications that promote transcription and global histone loss, inducing chromatin relaxation and the misregulation of gene expression. Such epigenetic changes are present also in cells from prematurely ageing individuals and likely contribute to the progression of the associated diseases (Lopez-Otin et al., 2013). In addition, the downregulation of chromatin remodelling factors, such as HP1α (heterochromatin protein 1 alpha), polycomb proteins and the NuRD complex, occurs during normal ageing and leads to global heterochromatin loss (Pollina and Brunet, 2011). In the context of premature-ageing syndromes, the absence of WRN (in WS) or the accumulation of progerin (in HGPS) also causes loss of heterochromatin and telomere attrition (Shumaker et al., 2006; Zhang et al., 2015). Likewise, cells from NGPS individuals exhibit profound changes in chromatin organization (Loi et al., 2016). In cells from individuals with HGPS, the epigenetic mark H3K27me3 is lost on the inactive X chromosome of affected females, as a consequence of downregulation of EZH2 (enhancer of Zeste homolog 2), the methyltransferase responsible for this mark. HGPS cells also exhibit the downregulation of H3K9me3 but increased levels of H4K20me3, which are epigenetic modifications that are associated with global repression of transcription (Shumaker et al., 2006). However, in a different study, increased levels of H3K9me3 were linked to accelerated senescence and compromised genome maintenance (Liu et al., 2013).

Sirtuin 1 (SIRT1) is thought to contribute to telomere maintenance through deacetylation of epigenetic marks such as H4K16ac (Palacios et al., 2010). By contrast, low levels of acetylation at H4K16 have been shown to correlate with a defective DNA-damage response (DDR) and double-strand-break repair to ionizing radiation (Sharma et al., 2010). In line with this, *Zmpste24^−/−^* mice show hypoacetylation of histones H2B and H4 (Osorio et al., 2010), likely due to the diminished association of the histone acetyltransferase Mof (male absent on the first) with the nuclear matrix. Rescue experiments performed either by Mof overexpression or by histone deacetylase inhibition promoted repair protein recruitment to DNA damage sites and substantially ameliorated aging-associated phenotypes in these mice (Krishnan et al., 2011).

Heterochromatin alterations have also been observed in other syndromes, such as an active heterochromatinization process mediated by SIRT1 in XP or CS cells after UV irradiation (Velez-Cruz et al., 2013), and the presence of unstable heterochromatin in FA cells (Edelman and Lin, 2001). Although epigenetic changes have not yet been identified in all progeroid syndromes, the frequent occurrence of both epigenetic and chromatin alterations tempt us to speculate that such defects are general features of these diseases.

### Aberrant nuclear architecture

Several progeroid syndromes−including HGPS, NGPS, APSs, RD and MAD−are associated with significant perturbations in nuclear organization and loss of nuclear envelope stability. In the case of HGPS, the presence of the farnesyl group in progerin, owing to the aberrant processing of lamin A, leads to progerin association with the inner nuclear membrane, generating defects in nuclear shape (Fig. 1) (Goldman et al., 2004). Such features are also observed in healthy ageing individuals owing to the sporadic activation of the same cryptic splice site in *LMNA* as in HGPS, whereas inhibition of this splice site reverses the nuclear defects associated with ageing (Righolt et al., 2011; Scaffidi and Misteli, 2006). Several studies have suggested that alterations in the nuclear lamina might lead to premature ageing by affecting the transcription profile of adult epithelial and mesenchymal stem cells and thereby interfering with their ability to retain an undifferentiated state (Espada et al., 2008; Scaffidi and Misteli, 2008). Progeroid fibroblasts obtained from NGPS individuals also show profound abnormalities in the nuclear lamina (Fig. 1) that can be rescued by the ectopic expression of wild-type *BANF1* (Puente et al., 2011; Soria-Valles et al., 2015a). Furthermore, cells from individuals with RD show an abnormal nuclear shape and heterogeneous deposits of unprocessed prelamin A (Columbaro et al., 2010). Severe nuclear morphological abnormalities are also observed in MAD and APS cells (Barthelemy et al., 2015). Collectively, these findings highlight the relevance of nuclear lamina aberrations as important features of different progeroid syndromes.

### Defects in cell cycle and mitosis

Cell cycle regulation is critically important for repairing genetic damage and preventing uncontrolled cell division. Changes in cell cycle dynamics, such as upregulation of p16 and other cell cycle inhibitors, are observed during physiological ageing and cellular senescence, and consequently represent valuable markers for the ageing process (Coppe et al., 2011). Nuclear envelope changes during cell division play a major role in the control of cell cycle progression (Camozzi et al., 2014). Accordingly, the targeting of nuclear lamina components into daughter cell nuclei in early G1 and cytokinesis progression is impaired in HGPS cells owing to the abnormal processing of lamin A (Dechat et al., 2007). FA proteins are essential for arresting the cell cycle until DNA damage is restored; thus, cells from FA patients have defects in DNA repair (Bogliolo and Surralles, 2015). Individuals with SS display an impaired G2/M checkpoint arrest and an increased number of centrosomes in mitotic cells (Griffith et al., 2008). Moreover, insufficiency of BubR1, a key mitotic checkpoint protein, causes aneuploidy, short lifespan, cachectic dwarfism, cataracts and other progeroid features (Baker et al., 2004).

### Cellular senescence

Cellular senescence is a state of stable cell cycle arrest and loss of replicative capacity that mainly results from DNA damage, oxidative stress and telomere shortening (van Deursen, 2014). In HGPS cells, progerin-induced senescence has been partially linked to p53 activation, a feature that is recapitulated in mouse models of this progeroid syndrome (Varela et al., 2005). Cells from NGPS patients also show premature cellular senescence characterized by markers such as senescence-associated β-galactosidase (SA-β-gal) staining, senescence-associated heterochromatin foci, increased levels of p16 and significant loss of lamin B1 (Soria-Valles et al., 2015a). Individuals affected by APS also present senescence phenotypes, driven by a dramatic reduction in lamin B1 expression (Bercht Pfleghaar et al., 2015). Likewise, fibroblasts from WS and SS patients show premature cellular senescence induced by replicative stress and faulty DNA repair, primarily due to the activation of stress kinase p38 (Davis et al., 2007; Tivey et al., 2013). Increased SA-β-gal staining and higher expression of p16 and p21 are also observed in *BLM*-, *WRN-* and *RECQL4-*depleted human fibroblasts (Lu et al., 2014). Furthermore, cells from *Xpc^−/−^* mice show increased β-galactosidase activity and high levels of progerin, p16 and ROS (Hosseini et al., 2015). Moreover, a mouse model of SS, characterized by a severe deficiency in ATR, shows accelerated ageing that is further aggravated by the loss of p53 (Murga et al., 2009). Hematopoietic stem cell senescence has also been reported in DC and FA (Stockklausner et al., 2015; Zhang et al., 2007).

### Metabolic defects

The ageing process is accompanied by many metabolic alterations, such as insulin resistance and physiological decline in growth hormone (GH), insulin-like growth factor-1 (IGF-1) and sex steroids. Accordingly, the insulin and IGF-1 signalling (IIS) pathway is extensively involved in ageing (Lopez-Otin et al., 2013). Moreover, dietary restriction (DR) increases healthspan in many organisms, further supporting a role for metabolism in ageing (Barzilai et al., 2012). Progeroid syndromes are also accompanied by metabolic changes. *Zmpste24^−/−^* mice exhibit profound transcriptional alterations in circulating levels of GH and IGF-1 (Marino et al., 2010), as well as changes in metabolic pathways that are associated with autophagy induction (Marino et al., 2008). Metabolic alterations are also present in progeroid syndromes that feature defects in DNA repair. *Wrn*-deficient mice exhibit hypertriglyceridemia and insulin resistance (Lebel and Leder, 1998). *ERCC1-*null mice show a shift toward anabolism and decreased GH-IGF-1 signalling (Niedernhofer et al., 2006), whereas CS mouse models show systemic suppression of the GH-IGF-I somatotropic axis (Box 1), increased antioxidant responses and hypoglycaemia (van der Pluijm et al., 2007). These studies suggest that unrepaired DNA damage induces a highly conserved metabolic response that is mediated by the IIS pathway, which redistributes resources from growth and proliferation for the preservation and protection of somatic integrity (Liao and Kennedy, 2014; Schumacher et al., 2009). Furthermore, mice with telomere dysfunction exhibit a marked deficiency in IGF-I-mTOR signalling, impaired energy homeostasis and suppressed mitochondrial biogenesis (Missios et al., 2014). Notably, mitochondrial dysfunction has been linked to the pathogenesis of progeroid syndromes such as HGPS and CS (Rivera-Torres et al., 2013; Scheibye-Knudsen et al., 2013). Cells derived from HGPS patients show a marked downregulation of mitochondrial oxidative phosphorylation proteins, accompanied by mitochondrial dysfunction (Rivera-Torres et al., 2013). These metabolic and mitochondrial alterations have inspired the development of different strategies for the treatment of progeroid syndromes (discussed below).

### Inflammation

Chronic inflammation is associated with normal and pathological ageing (Lopez-Otin et al., 2013). In line with this, a pro-inflammatory phenotype, named ‘inflammageing’, has been observed in mammals during ageing (Salminen et al., 2012). Moreover, senescent cells exhibit a senescence-associated secretory phenotype (SASP) characterized by the secretion of increasing levels of factors that alter their microenvironment in a paracrine manner, reinforcing senescence and activating immune surveillance. This phenomenon is mainly regulated by NF-κB (Acosta et al., 2013; Chien et al., 2011). We have recently found that the aberrant activation of NF-κB by ATM in mouse models of progeroid laminopathies induces the overexpression of pro-inflammatory cytokines and contributes to the pathogenesis of these syndromes (Osorio et al., 2012). Likewise, knocking down *Nfkb1* in mice causes premature ageing. Accordingly, the accumulation of senescent cells in *Nfkb1*^−/−^ tissues is blocked by the administration of anti-inflammatory drugs to these mice (Jurk et al., 2014). A recent study has also demonstrated that age-associated NF-κB hyperactivation impairs the generation of iPSCs by evoking the reprogramming repressor DOT1L, which promotes senescence signals and downregulates pluripotency genes (Soria-Valles et al., 2015a). An inflammatory phenotype associated with premature ageing has also been linked to WS (Goto et al., 2012). Similarly, mouse models of FA show inflammation-mediated upregulation of Notch signalling, which correlates with a decreased self-renewal capacity of FA HSCs (Du et al., 2013).

### Stem cell exhaustion

The ageing process is accompanied by a decrease in tissue regeneration and homeostasis, due to a decline in stem cell functions as a consequence of DNA damage, changes in tissue environment and alterations in tumour suppressor gene expression (Behrens et al., 2014). Because stem cells regenerate many adult tissues, changes in these cells likely contribute to the development of age-related diseases and to accelerated ageing syndromes. Consistent with this, progerin accumulation reduces the proliferative and differentiation capacity of pluripotent and multipotent (Box 1) mouse and human cells (Pacheco et al., 2014; Rosengardten et al., 2011). Furthermore, muscle-derived stem/progenitor cells (MDSPCs) from old and progeroid mice have proliferation and differentiation defects, whereas intraperitoneal administration of MDSPCs from young wild-type mice to progeroid mice confers a significant extension of lifespan and health via the secretion of certain factors that act systemically (Lavasani et al., 2012). Hematopoietic stem cell senescence has been described in the context of many progeroid syndromes. For example, *TERT* mutations that cause DC lead to cellular senescence and to the loss of CD34+ hematopoietic stem cells (Stockklausner et al., 2015). Moreover, TNFα induces premature senescence in bone marrow HSCs and in other tissues of FA mouse models. This induction correlates with ROS accumulation and oxidative DNA damage (Zhang et al., 2007). Together, these studies demonstrate the close relationship between progeroid syndromes and stem cell exhaustion, highlighting the importance of using cellular models to further understand the underlying pathobiology of these diseases and to guide the development of appropriate therapies.

## Therapeutic and rejuvenation strategies

Our improved understanding of the molecular basis of the progeroid syndromes has guided the development of therapeutic strategies for these disorders, particularly for HGPS (Gordon et al., 2014). Because progerin is permanently farnesylated, the first therapeutic strategy for treating HGPS involved the use of farnesyltransferase inhibitors (FTIs), such as lonafarnib, which has been used as a potential anticancer drug and had tolerable side effects in children. However, lonafarnib led to only limited improvements of symptoms in HGPS patients (Gordon et al., 2012), in part because, following FTI treatment, progerin is geranylgeranylated and gives rise to another toxic form of prelamin A (Varela et al., 2008). Consequently, an efficient blockade of progerin farnesylation must prevent both farnesylation and geranylgeranylation modifications, which provides the rationale behind a combined therapeutic approach that uses statins and aminobisphosphonates (Varela et al., 2008). This strategy has been successfully tested in *Zmpste24^−/−^* mice and is currently being evaluated for the treatment of individuals with HGPS (clinical trials: NCT00425607, NCT00879034 and NCT00916747).

Since these first strategies for HGPS treatment were described, additional therapies have been tested, with promising results, in mouse and cellular models of premature ageing. Many of these interventions derive from recent anti-ageing approaches designed to address alterations in cellular processes, such as nutrient-sensing, mitochondrial efficiency and autophagy, which are, as highlighted above, also deficient in progeria (de Cabo et al., 2014; Lopez-Otin et al., 2013). One such approach is treatment with the mTOR inhibitor rapamycin, which can suppress nuclear structure defects and postpone senescence in HGPS fibroblasts *in vitro* by inducing progerin clearance through autophagy (Cao et al., 2011). Additionally, the SIRT1 activator resveratrol rescues adult stem cell decline, slows down body weight loss and extends lifespan in *Zmpste24^−/−^* progeroid mice (Liu et al., 2012). Likewise, the administration of IGF-I to these mice has proven to positively affect both their health and lifespan (Marino et al., 2010).

Beyond these metabolic interventions, and because HGPS is caused by the activation of an alternative splice site, RNA therapy is another option for the treatment of progeria. Morpholino antisense oligonucleotides targeted to the altered splice site were first applied in HGPS fibroblasts to reduce progerin synthesis (Scaffidi and Misteli, 2005). This approach was also successfully used in the *Lmna^G609G^* HGPS mouse model, in which it led to a significant lifespan extension (Osorio et al., 2011). More recently, DOT1L inhibitors have proven to be effective as a rejuvenation strategy for both physiologically aged and HGPS and NGPS progeroid human cells (Soria-Valles et al., 2015a), and the treatment of *Zmpste24^−/−^* mice with DOT1L inhibitors increases their longevity and ameliorates their progeroid phenotypes (Soria-Valles et al., 2015a). Moreover, treatment of HGPS cells with a small molecule called ‘remodelin’, which targets the acetyltransferase NAT10, improves nuclear architecture and overall fitness of these progeroid cells (Larrieu et al., 2014). Other potentially beneficial HGPS treatments studied in cell-based and animal models include the administration of sodium salicylate, which inhibits the IκB kinase (IKK) complex (Osorio et al., 2012), pyrophosphate (Villa-Bellosta et al., 2013), methylene blue (Xiong et al., 2016) and Icmt inhibitors (Ibrahim et al., 2013).

Treatment with rapamycin has also been proposed as a therapeutic strategy for MAD because it efficiently triggers lysosomal degradation of farnesylated prelamin A (Camozzi et al., 2014). Resveratrol has proven to be efficient in reversing some of the clinically relevant phenotypes in *Wrn-*deficient mice, such as insulin resistance and liver steatosis, although it did not improve hypertriglyceridemia or inflammatory stress, nor extend the lifespan of these mice. Resveratrol-treated mutant mice also exhibited an increase in the frequency of different tumours (Labbe et al., 2011), and failed to show improvements in HGPS-associated bone defects (Strandgren et al., 2015). A high-fat diet rescues the metabolic, transcriptomic and behavioural phenotypes of a CS mouse model, whereas β-hydroxybutyrate, PARP (poly ADP ribose polymerase) inhibition and NAD^+^ supplementation can also rescue CS-associated phenotypes through activation of SIRT1, which is involved in cell cycle regulation and response to stressors (Scheibye-Knudsen et al., 2014). The reversal of mitochondrial defects in CS-derived cells using serine protease inhibitors has also been described (Chatre et al., 2015). Similarly, a glucose-enriched diet ameliorates the impaired energy homeostasis phenotype observed in mice with telomere dysfunctions, through the activation of glycolysis, mitochondrial biogenesis and oxidative glucose metabolism (Missios et al., 2014).

Recently, the clearance of senescent cells has been proposed as a promising strategy to promote healthy ageing, mainly through the administration of so-called ‘senolytic’ pharmacological agents that induce the death of senescent cells (Roos et al., 2016). Clearance of such cells in BubR1 hypomorphic progeroid mice delayed the onset of their ageing phenotype and attenuated its progression (Childs et al., 2015). Selective clearance of senescent cells was also shown to be an effective strategy for rejuvenation of tissue stem cells in normally ageing mice (Chang et al., 2016).

Read-through of premature termination codons in cells from WS and XP patients has been achieved using aminoglycosides: this restores WRN functionality in WS cells (Agrelo et al., 2015) and increases XPC protein production in XP cells (Kuschal et al., 2015). Genetic approaches have also been used to correct XP human cells (Dupuy and Sarasin, 2015), whereas genetic depletion of one allele of the p65 subunit of NF-κB or treatment with IKK inhibitors delays the age-related symptoms and pathologies of XFEPS (*XPF-ERCC1* progeroid syndrome) mice, which harbour mutations in *ERCC1* (involved in DNA excision repair) (Tilstra et al., 2012).

Another therapeutic option is the synthetic steroid danazol, which has anti-gonadotropic and anti-estrogenic activities and is capable of slowing down the progression of pulmonary fibrosis in individuals with DC (Zlateska et al., 2015). In the case of FA, treatment with p38 MAP-kinase inhibitors has improved the repopulating ability of *Fancc-*deficient HSCs (Saadatzadeh et al., 2009). Resveratrol also partially rescues the reduced repopulating ability of *Fancd2*-deficient HSCs (Zhang et al., 2010). Treating SS fibroblasts with p38 inhibitors can also restore their reduced replicative capacity *in vitro* and ameliorate their aged morphology (Tivey et al., 2013).

Importantly, recent studies suggest that the rate of ageing cannot only be modified by environmental and genetic factors, but also reversed (Freije and Lopez-Otin, 2012; Rando and Chang, 2012). Many rejuvenation strategies are based on epigenetic reprogramming, such as depletion of the methyltransferase Suv39h1, which reduces H3K9me3 levels, improves DNA repair capacity and delays senescence in progeroid cells, and also extends lifespan in progeroid mice (Ibrahim et al., 2013; Liu et al., 2013). In the future, strategies developed to treat progeroid syndromes could also be considered as treatments for pathologies associated with physiological ageing, as long as they share the same mechanisms. This would certainly expand the benefits of these discoveries to a wider medical community. Although many treatment options for individuals with ageing pathologies have been successfully tested in cellular and animal models, initiating therapeutic trials for rare diseases such as progeroid syndromes is an exceptionally difficult task because of the absence of longitudinal studies on different cohorts of patients. Also, the occurrence of only a low number of cases in any given country requires the setting up of clinical trials that follow identical protocols in different parts of the world (Osorio et al., 2011).

## Conclusions

Over recent years, advancements in our understanding of the genetic and molecular bases of premature aging disorders through the use of cellular and mouse models have led to a better understanding of the onset and progression of their clinical manifestations. On the basis of these studies, we have herein classified progeroid syndromes in two main categories, depending on the key molecular mechanisms involved. This classification scheme could provide a framework for the better understanding of the aetiology, biology and pathogenesis of progeroid syndromes. We have also appraised the latest findings made through the use of *in vitro* and *in vivo* models, which have helped to elucidate the processes that contribute to pathological and physiological ageing, and to characterize the cellular and organismal phenotypes of progeroid syndromes. Consequently, we have proposed nine hallmarks that characterize most progeroid syndromes, which extensively correlate with the nine hallmarks of ageing recently proposed (Lopez-Otin et al., 2013). Such hallmarks compile the most notable characteristics of progeroid syndromes and define the mechanisms underlying their pathogenesis, which might contribute to laying the groundwork for future studies on physiological and pathological ageing.

Finally, we have highlighted emerging therapies that could help to ameliorate the accelerated ageing phenotypes that underpin progeroid syndromes. It is well known that setting up of therapeutic trials for rare diseases is a difficult task owing to the lack of clinical correlational studies with similar evaluation guidelines and sufficient cohorts of patients (Osorio et al., 2011). This can present an obstacle to the testing of therapeutic approaches developed based on findings in cell- and animal-based studies. Nonetheless, a deeper characterization of the genetics and mechanisms underlying progeroid syndromes might enable earlier diagnosis and a better understanding of how the disease will progress in specific individuals, providing opportunities for earlier intervention and personalized treatment. Moreover, research focused on delaying or ameliorating physiological ageing could also contribute to developing a suitable therapeutic approach for progeroid syndromes.
